# Correlates and Predictors of PTSD Symptoms Among Healthcare Workers During the COVID-19 Pandemic: Results of the egePan-VOICE Study

**DOI:** 10.3389/fpsyt.2021.686667

**Published:** 2021-08-17

**Authors:** Susann Steudte-Schmiedgen, Lisa Stieler, Yesim Erim, Eva Morawa, Franziska Geiser, Petra Beschoner, Lucia Jerg-Bretzke, Christian Albus, Nina Hiebel, Kerstin Weidner

**Affiliations:** ^1^Department of Psychotherapy and Psychosomatic Medicine, Faculty of Medicine, Technische Universität Dresden, Dresden, Germany; ^2^Department of Psychosomatic Medicine and Psychotherapy, University Hospital of Erlangen, Friedrich-Alexander University Erlangen-Nürnberg (FAU), Erlangen, Germany; ^3^Department of Psychosomatic Medicine and Psychotherapy, University Hospital Bonn, Bonn, Germany; ^4^Department of Psychosomatic Medicine and Psychotherapy, Ulm University Medical Center, Ulm, Germany; ^5^Department of Psychosomatics and Psychotherapy, University Hospital of Cologne, Cologne, Germany

**Keywords:** COVID-19, post-traumatic stress disorder, depression, anxiety, healthcare, mental health

## Abstract

**Background:** The COVID-19 pandemic has led to ongoing challenges for healthcare systems across the world. Previous research has provided evidence for an increased prevalence of depression and anxiety as well as post-traumatic stress disorder (PTSD). In Germany, however, only scarce data on correlates and predictors for PTSD symptomatology in the context of the COVID-19 pandemic among healthcare workers (HCW) are available.

**Methods:** This research is part of a large prospective web-based survey (egePan-VOICE study) among HCW in Germany. The current sample (*N* = 4,724) consisted of physicians (*n* = 1,575), nurses (*n* = 1,277), medical technical assistants (MTA, *n* = 1,662), and psychologists (*n* = 210). PTSD symptomatology was measured using the abbreviated version of the Impact of Event Scale (IES-6). In addition, sociodemographic, occupational, COVID-19-related, psychological (e.g., depressive symptoms and generalized anxiety), as well as work-related variables were assessed.

**Results:** Our findings revealed significant higher PTSD symptoms with medium effect sizes among HCW reporting an increased self-report burden during the pandemic, increased fear of becoming infected or infecting relatives with the virus, sleep problems, feeling physically or mentally exhausted, as well as increased levels of depressiveness and generalized anxiety. According to multiple linear regression analysis, the most relevant predictors for higher IES-6 scores were increased level of generalized anxiety and depressiveness, increased fear of infecting relatives, as well as medical profession (MTA compared to physicians).

**Conclusion:** Despite the cross-sectional design of our study, the here identified associations with PTSD symptomatology may provide a basis for future preventive interventions.

## Introduction

Besides having adverse effects on physical health, the COVID-19 pandemic has been associated with an increase in psychological distress [i.e, emotional suffering related to stressors and demands that are difficult to cope with in daily life; (1)] and detrimental effects on mental health and well-being. This has been documented by increasing evidence across the world [e.g., (2–4)]. In Germany, previous studies examining the general population indicated high prevalence rates of 25–45% for generalized anxiety, 14–25% for depression, 65% for psychological distress, and 59% for COVID-19-related fear (5, 6). As the COVID-19 pandemic is associated with ongoing challenges for healthcare systems with healthcare workers (HCW) being regularly confronted with potential infection, illness, and death, research efforts have been made to investigate mental health burden in this population [for meta-analyses, see (7–9)].

Interestingly, a recent study by our work group (10) has revealed that during the first wave of the pandemic, HCW working in hospitals showed a lower burden of mental distress compared to the general population, suggesting an adequate coping capacity in this population. Specifically, the prevalence rate of depression and anxiety was 17.4 and 17.8% for physicians, 21.6 and 19.0% for nurses, and 23.0 and 20.1% for medical technical assistants (MTA), respectively. Hence, these data support the notion that medical, laboratory, radiology, or pharmaceutical-technical assistants (referred to as MTA) were among the most vulnerable group in this context [see also (11)]. Our findings further indicated that higher scores of depressive symptoms were related to insufficient recovery during leisure time, increased alcohol consumption, and less trust in colleagues, while elevated anxiety scores were additionally associated with increased fear of becoming infected with COVID-19. Furthermore, we revealed that higher levels of social support and optimism were related to lower indices of depression and generalized anxiety, respectively, and that these psychosocial resources were more relevant than other sociodemographic or occupational characteristics (12).

Since the COVID-19 pandemic is considered a traumatic stressor (13), previous research has also investigated its impact on post-traumatic stress disorder (PTSD) symptomatology besides focusing on depression and unspecific anxiety as well as general stress. PTSD is one possible psychological consequence of trauma experience and is characterized by symptoms such as recurrent intrusions, avoidance, and hyperarousal (14). In the context of the COVID-19 pandemic, most of previous evidence suggests higher prevalence rates of PTSD or PTSD symptom severity in HCW as compared to the general population during pandemics including COVID-19 [e.g., (15–18)] with a wide range of prevalence rates [7.4–35%; e.g., (18–23)]. Most of these studies further investigated determinants as well as risk and/or protective factors for increased PTSD symptomatology. Regarding sociodemographic and occupational characteristics, previous evidence suggests that higher PTSD symptoms were associated with female gender and younger age (15, 18), a higher exposure level [e.g., high risk wards and front line setting; (15, 18, 19, 22)], medical profession of a nurse (15, 18, 19, 24, 25), and fewer years of work experience (15, 18). Further, a poor psychological status as evidenced by higher levels of depression, anxiety, stress symptoms, and poor sleep quantity or quality was found to be predictive of increased PTSD levels [e.g., (18, 20, 21)]. On the other hand, high social support was identified as a protective factor for PTSD symptoms among HCW (15). Further, a recent study revealed that besides low emotional support, burnout symptoms and health anxiety were specifically related to PTSD symptomatology (22). The latter finding is in line with Wang et al. (19) suggesting that not worrying about infection was a protective factor for PTSD symptomatology. Besides psychological characteristics, previous evidence emerged suggesting that work-related factors were associated with the degree of PTSD symptoms. For instance, low job satisfaction was identified as a major risk factor for PTSD (18), while high work support and a good job organization were defined as protective factors (15). Furthermore, in another study among medical staff, a good collegial relationship was related to lower PTSD scores (20). Wang et al. (19) further revealed that lacking confidence in protection measures was related to higher PTSD levels. Taken together, increasing evidence emerged to elucidate knowledge on prevalence rates and predictors of PTSD symptoms in HCW during COVID-19, with most of the above studies stemming from Asia and Europe. However, in Germany, only scarce data on correlates and predictors for PTSD symptomatology in this population of interest are available.

Hence, the aim of the current study was to examine variables contributing to increased PTSD symptomatology during the COVID-19 pandemic among German HCW. For this, we investigated cross-sectional data of the first wave of the pandemic as part of the prospective web-based egePan-VOICE survey. This study, which was conducted within the German cooperation network of university medicine (NUM) by the Ministry of Education and Research, allowed a detailed investigation of correlates and predictors of PTSD symptomatology in a large sample of HCW. Specifically, we aimed to address the following hypotheses:

i) *Sociodemographic and occupational characteristics*: We hypothesized an older age, female gender, less work experience to be related to PTSD. Furthermore, we set out to examine whether an increased burden due to working in fulltime or working in an inpatient setting was related to higher PTSD symptoms. Concerning medical profession, we aimed to investigate whether our previous finding of increased depressiveness and generalized anxiety among MTA (10) would also generalize to PTSD symptoms, followed by nurses.
ii) *COVID-19-related characteristics*: We hypothesized that an increased closeness to infected patients or contaminated material was related to higher PTSD symptoms. Further, we aimed to investigate whether our finding of increased depression and anxiety symptoms in HCW belonging to a risk group or who had been displaced of the department due to the pandemic (12) was also evident for elevated PTSD scores.
iii) *Psychological characteristics:* We hypothesized that a poorer psychological status as reflected by an increased overall burden during the pandemic, increased level of depressiveness, general anxiety, fear of infection, sleeping problems, physical/mental exhaustion, smoking, and alcohol consumption was associated with higher PTSD symptoms. Further, we expected that a high level of social support should co-occur with lower PTSD symptoms.
iv) *Work-related characteristics:* We hypothesized that a higher occupancy rate, insufficient staff, insufficient recovery during leisure time, less trust in colleagues, increased burden by the increase of workload and/or change in duties, and insufficient protective measures during COVID-19 as well as increased chronic work-related stress were associated with higher PTSD symptoms.

Finally, we set up to identify the most relevant predictors for PTSD symptomatology.

## Methods

### Participants and Procedures

The current study is part of the prospective study “VOICE,” which is an online survey in order to assess stressors and resources of HCW during the COVID-19 pandemic. Here, we specifically focus on cross-sectional data of the first wave being collected between April 20 and July 5, 2020, using a self-report online questionnaire *via* SoSci Survey and Unipark. Participants were mainly recruited among medical staff of the university hospitals of Erlangen, Bonn, Ulm, Cologne, and Dresden. Specifically, the psychosomatic departments of the respective university hospitals distributed the link *via* online platforms or mailing lists. In addition, other hospitals and professional networks supported the study and shared the link. The online survey comprised 77 items and took 15 min to complete. General inclusion criteria were defined as a minimum age of 18 years, working in the healthcare sector, residence/working place in Germany, as well as sufficient German language skills.

In the online survey, 8,071 HCW with diverse medical professions took part. To ensure homogeneity of the sample and comparability with previous research in this context, we further defined the following specific inclusion criteria for the current study: belonging to a medical profession of physician (*n* = 1,575, 33.3%), nurse (*n* = 1,277, 27.0%), MTA (*n* = 1,662, 35.2%), or psychologist (*n* = 210, 4.4%) as well as working in university hospitals and other hospitals of maximum care (*n* = 3,806, 80.6%) or in an outpatient setting (*n* = 918, 19.4%). Accordingly, 3,347 individuals were excluded who could not be clearly assigned to one of the above professional groups or to either the inpatient or outpatient setting, resulting in a total sample of 4,724 HCW included in the current study.

### Measures

#### Sociodemographic and Occupational Characteristics

The online questionnaire assessed sociodemographic variables (i.e., age and gender) and occupational characteristics including work setting, medical profession, years of professional experience, and employment status (full-time vs. part-time).

#### PTSD Symptoms

PTSD symptoms were assessed by the abbreviated six-item version of the Impact of Event Scale [IES-6; (26, 27)]. Specifically, the IES-6 consists of the following items reflecting PTSD symptom clusters (i.e., intrusion, avoidance/numbing, and hyperarousal): “I thought about it when I didn't mean to,” “I felt watchful or on-guard,” “Other things kept making me think about it,” “I was aware that I still had a lot of feelings about it, but I didn't deal with them,” “I tried not to think about it,” “I had trouble concentrating.” In our study, participants were asked to think about the COVID-19 pandemic and if any of the abovementioned reactions occurred during the last 2 weeks. The response categories of the original IES were used (not at all = 0, rarely = 1, sometimes = 3, and often = 5). The total IES-6 score ranges from 0 to 30. Thoresen et al. (26) supported the validity of the IES-6 revealing a Cronbach's Alpha of 0.80. In the current sample, we detected a Cronbach's Alpha of 0.70.

#### COVID-19-Related Variables

Information on COVID-19-related variables including displacement of the department due to the pandemic, having direct contact at work with COVID-19 infected patients, contact with contaminated material during work, belonging to an at-risk group due to age or a chronic illness, and being infected with the Sars-Cov-2 virus were obtained.

#### Psychological Characteristics

The overall burden during the COVID-19 pandemic was measured with one item on a scale from 0 “not at all” to 4 “very strong,” respectively. Specifically, individuals were asked “How much burden have you felt due to the COVID-19 pandemic over the last 2 weeks.” Depressive symptoms and unspecific anxiety symptoms over the past 2 weeks were assessed using the two-item Patient Health Questionnaire (PHQ-2) and the two-item Generalized Anxiety Module (GAD-2), respectively. Participants rated the four items on a scale from 0 = “not at all” to 3 = “nearly every day.” The respective sum scores range from 0 to 6. A cut-off value of ≥3 on the two subscales is assumed to indicate cases of depression and anxiety, respectively. Previous evidence has supported the reliability and validity of the PHQ-2 and GAD-2 [Cronbach's Alpha values > 0.80; (28)]. In the present sample, Cronbach's Alpha scores of 0.76 for the PHQ-2 and 0.78 for the GAD-2 were revealed.

The ENRICHD Social Support Inventory [ESSI; (29)] was applied to measure social support. The ESSI is a five-item questionnaire with a score ranging from 5 to 25. A cut-off value of ≤18 and the answer of at least two items ≤3 are indicative of low social support (30). Kendel et al. (29) reported a Cronbach's Alpha of 0.89 for the ESSI, which is in line with the Cronbach's Alpha score of the current sample.

In addition, anxiety about infection, sleep problems, physical or mental exhaustion, smoking, and drinking alcohol during the COVID-19 pandemic over the past 2 weeks were measured using six items [based on (31)] on a scale from 0 = “strongly disagree” to 4 = “strongly agree” (for specific items, see **Table 3**).

#### Work-Related Characteristics

Working conditions and potential work problems during the COVID-19 pandemic over the last 2 weeks were assessed with seven items on a scale from 0 = “strongly disagree” to 4 = “strongly agree” with regards to the degree of occupancy of the wards, sufficient staff, sufficient recovery during leisure time, trust in colleagues, workload, changes of duties, as well as feeling of being protected (for specific items, see **Table 4**). The items were selected based on Matsuishi et al. (31).

To assess chronic work-related stress, the effort-reward imbalance questionnaire was used [ERI; (32)]. This instrument assesses the balance between work-related efforts and rewards received. Previous evidence has consistently shown that a non-reciprocity (i.e., high efforts spent and low rewards received) contributes to deleterious mental and somatic health [e.g., (33, 34)]. The 10 items of the ERI that were used in this study were rated on a scale from 1 to 4. The effort scale consists of three items resulting in a total score ranging from 3 to 12, and the reward scale consists of seven items resulting in a total score ranging between 7 and 28. The ERI ratio (i.e., total score effort/total score reward*correction factor 3/7) was calculated. A cut-off value of ≥1 is reflective of a non-reciprocity between (too much) efforts and (too little) reward (32, 35). The ERI is a psychometrically well-justified measure (36). In the present sample, Cronbach's Alpha scores of 0.74 for the effort scale and 0.75 for the reward scale were revealed.

### Statistical Analyses

Statistical analyses were performed using SPSS for Windows, version 27 (IBM, Chicago, Illinois). Statistical tests were two-tailed and based on an alpha-level of significance of 0.05. The expectation-maximization algorithm was applied to complete missing data. Univariate analyses of variance and *t*-tests were conducted to examine group differences in PTSD symptomatology with regard to sociodemographic, occupational, and COVID-19-related variables. The effect sizes η_p_^2^ (partial eta-squared) and Cohen's d following Cohen (37) are also reported (η_p_^2^ ≥ 0.01 = small, η_p_^2^ ≥ 0.06 = medium, η_p_^2^ ≥ 0.14 = large effect; *d* ≥ 0.2 = small, *d* ≥ 0.5 = medium, and *d* ≥ 0.8 = large effect). Results were followed up by Bonferroni-corrected *post-hoc* analyses if required. For the continuous psychological and work-related characteristics, bivariate correlation analyses were conducted. Pearson correlation coefficient following Cohen (37) is reported (| *r* | > 0.10 = small, | *r* | > 0.30 = moderate, | *r* | > 0.50 = large effect). In order to examine the predictive value of the variables, which emerged as significant in the univariate analyses of variance, *t*-tests, or correlation analyses, multiple linear regression analysis with the forced entry method was performed. The variable “patient care” was not included in the regression analysis due to a high intercorrelation with the variable work experience. Variance inflation factors of the respective variables were below 10, indicating no issues of multicollinearity (see **Table 5**).

## Results

### Description of the Study Population

Tables 1, 2 illustrate socio-demographic, occupational, and COVID-19-related characteristics of the study sample (*N* = 4,724). Three quarters of the sample comprised of women (74.5%) and less than half of the sample were younger than 40 years (44.9%). The majority of the sample reported to have professional experience in patient care of more than 6 years (77.9%). Two-thirds (66.9%) of participants indicated to work in full-time. 51.3% had direct contact with COVID-19 infected patients and 51.4% had contact with contaminated material. 28.6% of the sample reported to belong to a risk group because of their age and/or previous illness. Only 1.1% of the sample indicated a previous infection with the virus. Concerning psychological variables, 41.7% of the sample reported a strong or very strong current burden due to the COVID-19 pandemic. Prevalence rates of 19.9 and 20.4% were revealed for suspected depression and generalized anxiety, respectively. Of the total sample, 22.7% reported a low level of social support (ESSI ≤ 18). Concerning chronic work-related stress, high work-related efforts spent and low rewards received (ERI ratio ≥ 1) were reported by 55.1%.

**Table 1:** Group differences in PTSD symptoms with regards to sociodemographic and occupational characteristics among the sample of healthcare workers (*N* = 4,724).

	***n* (%)**	**IES-6 total mean (SD)**	**Test statistics**	***p***	**Effect size**
**Gender**			*t*_4704_ = 9.678^**a**^	<0.001^**a**^	*d* = 0.331^**a**^
Women	3,520 (74.5)	16.3 (5.7)			
Men	1,186 (25.1)	14.4 (6.0)			
Divers	18 (0.4)	12.0 (7.4)			
**Age, years**			*F*_4,4,719_ = 11.427	<0.001	*$\eta_{p}^{2}$* = 0.010
18–30	952 (20.2)	15.3 (5.6)			
31–40	1,167 (24.7)	15.1 (6.0)			
41–50	1,057 (22.4)	16.2 (6.0)			
51–60	1,227 (26.0)	16.5 (5.7)			
>60	321 (6.8)	15.9 (5.7)			
**Patient care^b^**			*t*_4,717_ = 4.044	<0.001	*d* = 0.190
Yes	4,209 (89.2)	15.7 (5.9)			
No	510 (10.8)	16.8 (5.6)			
**Professional experience in patient care^c^**			*F*_2,4,206_ = 9.967	<0.001	*$\eta_{p}^{2}$* = 0.005
<3 years	403 (9.6)	14.7 (5.9)			
3–6 years	527 (12.5)	15.1 (5.6)			
>6 years	3,279 (77.9)	15.9 (5.9)			
**Employment status^d^**			*t*_4,716_ = 6.631	<0.001	*d* = 0.174
Full-time	3,154 (66.9)	15.5 (5.9)			
Part-time	1,564 (33.1)	16.5 (5.8)			
**Medical profession**			*F*_3,4,720_ = 54.483	<0.001	*$\eta_{p}^{2}$* = 0.033
Physicians	1,575 (33.3)	14.8 (6.1)			
Nurses	1,277 (27.0)	15.7 (5.7)			
Medical technical assistants^*^	1,662 (35.2)	17.1 (5.4)			
Psychologists	210 (4.4)	13.5 (6.5)			
**Work setting**			*t*_4,722_ = 3.762	<0.001	*d* = 0.138
Hospital	3,806 (80.6)	15.6 (5.9)			
Outpatient	918 (19.4)	16.4 (5.8)			

*Test statistics: t-test (t-value) effect size: Cohen's d; ANOVA (F-value) effect size: partial η^2^*.

a *N = 4,706 (diverse was excluded from ANOVA)*.

b *N = 4,719*.

c *N = 4,209*.

d *N = 4,718*.

* *The group consisted of the following professional subgroups: Medical assistants, Medical-technical laboratory assistants, Medical-technical radiology assistants, Pharmaceutical-technical assistants*.

Post-hoc tests: **Age:** significant difference between 18–30*41–50, 18–30*51–60, 31–40*41–50, 31–40*51–60; **Professional Experience:** all comparisons are significant except <3 years*3–6 years; **Medical profession:** all comparisons are significant.

**Table 2:** Group differences in PTSD symptoms with regards to COVID-19-related characteristics among the sample of healthcare workers (*N* = 4,724).

	***n* (%)**	**IES-6 total mean (SD)**	**Test statistics**	***p***	**Effect size**
**Contact with infected patients^a^**			*t*_4,690_ = 2.378	0.017	*d* = 0.069
Yes	2,409 (51.3)	16.0 (5.8)			
No	2,283 (48.7)	15.6 (6.0)			
**Contact with contaminated material^b^**			*t*_4,684_ = 4.042	<0.001	*d* = 0.003
Yes	2,407 (51.4)	16.1 (5.8)			
No	2,279 (48.6)	15.4 (6.0)			
**Risk group because of age or previous illness^c^**			*t*_4,686_ = 8.424	<0.001	*d* = 0.272
Yes	1,343 (28.6)	16.9 (5.7)			
No	3,345 (71.4)	15.3 (5.9)			
**Infection with SARS-CoV-2 virus^b^**			*F*_2,4683_ = 1.199	0.302	*$\eta_{p}^{2}$* = 0.001
Yes	50 (1.1)	16.1 (6.2)			
No	2,544 (54.3)	15.7 (5.8)			
I don't know	2,092 (44.6)	15.9 (6.0)			
**Displacement of the department due to the pandemic^d^**			*t*_4,690_ = 2.695	0.007	*d* = 0.115
Yes	641 (13.7)	16.4 (5.6)			
No	4,051 (86.3)	15.7 (5.9)			

*Test statistics: t-test (t-value) effect size: Cohen's d; ANOVA (F-value) effect size: partial η^2^*.

a *N = 4,692*.

b *N = 4,686*.

c *N = 4,688*.

d *N = 4,692*.

### Correlates of PTSD Symptoms

#### Sociodemographic and Occupational Characteristics

Table 1 illustrates mean IES-6 scores respective of sociodemographic and occupational characteristics. Group differences were revealed for gender, age, working in patient care, professional experience in patient care, employment status, medical profession, and work setting. Specifically, increased IES-6 scores were reported by women, MTA, and HCW at an age between 41 and 60 years, however with only small effect sizes (*p*'s < 0.001, *d* = 0.331, 0.010 ≤ $\eta_{\text{p}}^{2}$ ≤ 0.033). Higher PTSD symptoms also emerged in HCW working not in patient care, working in part-time, having work experience of more than 6 years, as well as working in an outpatient setting, however with negligible effect sizes (*p*'s < 0.001, 0.138 ≤ *d* ≤ 0.190, $\eta_{\text{p}}^{2}$ = 0.005).

#### COVID-19-Related Characteristics

Table 2 illustrates mean IES-6 scores respective of COVID-19-related characteristics. Elevated PTSD scores were observed for HCW who belonged to a risk group because of age or a previous illness, with a small effect size (*p* < 0.001, *d* = 0.272). Higher IES-6 scores were also revealed for participants who had contact with infected patients or with contaminated material as well as HCW who were displaced of their department due to the pandemic, however with only negligible effect sizes (*p*'s ≤ 0.017, 0.003 ≤ *d* ≤ 0.115). No effect emerged with regards to an infection with the virus (*p* = 0.302).

#### Psychological Characteristics

Table 3 illustrates Pearson correlation coefficients between PTSD symptoms and psychological characteristics. Higher IES-6 scores were moderately related to higher levels of depression and anxiety as well as an increased burden during COVID-19, an increased fear of being infected with the virus, increased fear of infecting the family or relatives with the virus, having sleep problems, and suffering from physical or mental exhaustion (*p*'s < 0.001, 0.314 ≤ *r*'s ≤ 0.421). In addition, higher IES-6 scores were associated with a lower level of emotional support and drinking more alcohol during the COVID-19 pandemic with small effect sizes (*p* < 0.001, −0.124 ≤ *r*'s ≤ 0.107). Only a negligible association emerged between smoking and IES-6 scores (*p* < 0.001, *r* = 0.091).

**Table 3:** Correlations of PTSD symptoms with psychological characteristics among the sample of healthcare workers (*N* = 4,724).

	**IES-6**	**1**.	**2**.	**3**.	**4**.	**5**.	**6**.	**7**.	**8**.	**9**.
1. Overall burden during COVID-19 pandemic	0.346^***^									
2. PHQ-2 (depression)	0.421^***^	0.414^***^								
3. GAD-2 (anxiety)	0.387^***^	0.369^***^	0.656^***^							
4. ESSI (social support)	−0.124^***^	−0.140^***^	−0.288^***^	−0.283^***^						
5. I was afraid to become infected^a^	0.339^***^	0.321^***^	0.312^***^	0.211^***^	−0.085^**^					
6. I was afraid to infect relatives or my family^a^	0.314^***^	0.278^***^	0.262^***^	0.214^***^	−0.037^*^	0.623^***^				
7. I had trouble sleeping^b^	0.364^***^	0.403^***^	0.488^***^	0.455^***^	−0.203^***^	0.261^***^	0.226^***^			
8. I felt physically or mentally exhausted^b^	0.379^***^	0.536^***^	0.532^***^	0.532^***^	−0.212^***^	0.285^***^	0.262^***^	0.632^***^		
9. I smoked more^c^	0.091^***^	0.105^***^	0.125^***^	0.126^***^	−0.078^***^	0.096^***^	0.108^***^	0.141^***^	0.136^***^	
10. I drank more alcohol^d^	0.107^***^	0.146^***^	0.198^***^	0.204^***^	−0.070^***^	0.081^***^	0.085^***^	0.192^***^	0.176^***^	0.294^***^

*IES-6, Impact of Event Scale; PHQ-2, Patient Health Questionnaire; GAD-2, Generalized Anxiety Module; ESSI, ENRICHD Social Support Inventory. Overall burden during the COVID-19 pandemic (0 = not at all, 1 = little, 2 = middle, 3 = strong, 4 = very strong), Items 5–10 (1 = strongly disagree, 2 = rather disagree, 3 = neither agree nor disagree, 4 = rather agree, 5 = strongly agree). Significance threshold (two-tailed):*

* *p < 0.05*,

** *p < 0.01*,

*** *p < 0.001*.

a *N = 4,575*.

b *N = 4,573*.

c *N = 4,567*.

d *N = 4,569*.

#### Work-Related Characteristics

Table 4 illustrates Pearson correlation coefficients between PTSD symptoms and work-related variables. Chronic work-related stress, sufficient recovery during leisure time, sufficient trust in colleagues, feeling burdened by the increase of workload, and change in duties were found to be related to IES-6 scores with small effect sizes (*p*'s < 0.001, −0.105 ≤ *r*'s ≤ 0.281). Significant associations were also observed between IES-6 scores and occupancy rate, sufficient staff, and protection measures, however with negligible effect sizes (*p*'s < 0.001, −0.075 ≤ *r*'s ≤ 0.059).

**Table 4:** Correlations of PTSD symptoms with work-related characteristics during the COVID-19 pandemic among the sample of healthcare workers (*N* = 4,724).

	**IES-6**	**1**.	**2**.	**3**.	**4**.	**5**.	**6**.	**7**.
1. ERI ratio (chronic work-related stress)	0.258^***^							
2. Degree of occupancy of the wards^a^	0.059^***^	0.304^***^						
3. There is sufficient staff for the current work load^b^	−0.096^***^	−0.448^***^	−0.439^***^					
4. I can recover sufficiently during my free time^c^	−0.207^***^	−0.395^***^	−0.224^***^	−0.384^***^				
5. I can trust in my colleagues, when it gets difficult during work^d^	−0.105^***^	−0.282^***^	−0.052^**^	0.237^***^	0.292^***^			
6. I was burdened by the increase of workload.^e^	0.242^***^	0.506^***^	0.467^***^	−0.492^***^	−0.429^***^	−0.203^***^		
7. I was burdened by the change in my duties.^e^	0.281^***^	0.296^***^	0.090^***^	−0.168^***^	−0.294^***^	−0.130^***^	0.454^***^	
8. I felt protected by measures taken by national and local authorities^f^	−0.075^***^	−0.236^***^	−0.075^***^	0.163^***^	0.231^***^	0.170^***^	−0.161^***^	−0.176^***^

*IES-6, Impact of Event Scale; ERI, Effort-Reward Imbalance Questionnaire. Degree of occupancy of the wards (1 = strongly below average, 2 = slightly below average, 3 = average, 4 = slightly above average, 5 = strongly above average), Items 3–8 (1 = strongly disagree, 2 = rather disagree, 3 = neither agree nor disagree, 4 = rather agree, 5 = strongly agree). Significance threshold (two-tailed):*

* *p < 0.05*,

** *p < 0.01*,

*** *p < 0.001*.

a *N = 4,686*.

b *N = 3,894*.

c *N = 3,895*.

d *N = 3,849*.

e *N = 4,575*.

f *N = 4,574*.

### Multivariate Regression Analysis Predicting PTSD Symptom Severity

Table 5 shows results of multiple regression analysis including the following variables of interest: sociodemographic and occupational characteristics (i.e., gender, age, professional experience, employment status, work profession, and work setting), COVID-19-related characteristics (i.e., contact with infected patients, contact with contaminated material, risk group, and displacement of the department), psychological characteristics (i.e., burden during COVID-19, depressiveness, general anxiety, social support, fear of becoming infected, fear to infect family, sleep problems, physical or mental exhaustion, smoking, and alcohol consumption), and work-related characteristics (i.e., chronic work-related stress, occupancy rate, sufficient staff, sufficient recovery, trust in colleagues, workload, changes of duties, and protective measures). The final model explained 30.8% of the variance in IES-6 scores. In our model, the best predictors for higher IES-6 scores were an increased level of generalized anxiety (GAD-2; *p* < 0.001, β = 0.141), depressiveness (PHQ-2; *p* < 0.001, β = 0.136), increased fear of infecting family or relatives (*p* < 0.001, β = 0.112), and belonging to medical profession of MTA compared to physicians (*p* < 0.001, β = 0.103). Further variables predicting PTSD symptoms, but with a lower predictive value (standardized β < 0.1), were the following (in descending order): belonging to medical profession of MTA compared to psychologists (*p* < 0.001, β = 0.096), fear of becoming infected (*p* < 0.001, β = 0.092), having sleep problems (*p* < 0.001, β = 0.086), age of >40 years (*p* < 0.001, β = 0.078), increased burden during COVID-19 (*p* < 0.001, β = 0.077), increased change of duties (*p* < 0.001, β = 0.071), physical or mental exhaustion (*p* = 0.005, β = 0.064), working part-time (*p* < 0.001, β = 0.057), female gender (*p* = 0.001, β = 0.054), sufficient staff (*p* = 0.023, β = 0.042), working in an outpatient setting (*p* = 0.013, β = 0.040), and protective measures taken by national and local authorities (*p* = 0.025, β = 0.035). For the other variables of interest, no significant predictive effect emerged (*p*'s > 0.059).

**Table 5:** Linear regression analysis for severity of PTSD symptoms (IES-6) among the sample of healthcare workers (*N* = 3,342).

**Independent variables**	**IES-6 (** $R_{adj}^{2}$ **=** **0.308)**					
	**Regression coefficient (95% CI)**	**SE**	**Beta**	***T***	**VIF**	***p***
Constant	2.686 (0.569, 4.803)	1.080	−	2.487		0.013
**Sociodemographic and occupational characteristics**						
Gender (male vs. female)	0.739 (0.317, 1.162)	0.216	0.054	3.429	1.190	**0.001**
Age (≤40 vs. >40 years)	0.946 (0.512, 1.379)	0.221	0.078	4.278	1.592	** <0.001**
Professional experience	−0.188 (−0.514, 0.137)	0.166	−0.019	−1.136	1.412	0.256
Employment status (full- vs. part-time)	0.734 (0.344, 1.124)	0.199	0.057	3.692	1.163	** <0.001**
Physicians (ref. = MTA)	−1.303 (−1.763, −0.843)	0.234	−0.103	−5.558	1.669	** <0.001**
Nurses (ref. = MTA)	−0.456 (−0.929, 0.018)	0.241	−0.033	−1.887	1.481	0.059
Psychologists (ref. = MTA)	−2.618 (−3.495, −1.741)	0.447	−0.096	−5.853	1.296	** <0.001**
Work setting (hospital vs. outpatient)	0.585 (0.126, 1.044)	0.234	0.040	2.498	1.262	**0.013**
**COVID-19-related characteristics**						
Contact with infected patients (no vs. yes)	0.039 (−0.428, 0.507)	0.238	0.003	0.165	1.855	0.869
Contact with contaminated material (no vs. yes)	0.022 (−0.447, 0.492)	0.239	0.002	0.093	1.891	0.926
Risk group (no vs. yes)	0.340 (−0.071, 0.750)	0.209	0.025	1.623	1.174	0.105
Displacement of the department (no vs. yes)	0.395 (−0.108, 0.898)	0.257	0.023	1.538	1.090	0.124
**Psychological characteristics**						
Burden during COVID-19	0.463 (0.231, 0.695)	0.118	0.077	3.917	1.844	** <0.001**
PHQ-2 score	0.545 (0.379, 0.711)	0.085	0.136	6.435	2.151	** <0.001**
GAD-2 score	0.595 (0.423, 0.767)	0.088	0.141	6.796	2.074	** <0.001**
ESSI score	0.026 (−0.022, 0.074)	0.024	0.017	1.077	1.208	0.282
Fear of becoming infected^*^	0.433 (0.250, 0.615)	0.093	0.092	4.657	1.870	** <0.001**
Fear to infect family^*^	0.504 (0.334, 0.674)	0.087	0.112	5.814	1.805	** <0.001**
Sleep problems^*^	0.380 (0.214, 0.547)	0.085	0.086	4.470	1.781	** <0.001**
Physical or mental exhaustion^*^	0.304 (0.093, 0.515)	0.108	0.064	2.823	2.471	**0.005**
Smoked more^*^	0.048 (−0.122, 0.218)	0.087	0.009	0.554	1.175	0.580
Drank more alcohol^*^	−0.057 (−0.217, 0.104)	0.082	−0.011	−0.691	1.177	0.489
**COVID-19-related work conditions**						
ERI ratio	0.352 (−0.116, 0.820)	0.239	0.028	1.475	1.781	0.140
Degree of occupancy of the wards	−0.122 (−0.296, 0.052)	0.089	−0.024	−1.371	1.451	0.170
Sufficient staff^*^	0.194 (0.027, 0.360)	0.085	0.042	2.282	1.612	**0.023**
Sufficient recovery^*^	0.127 (−0.049, 0.303)	0.090	0.027	1.414	1.755	0.157
Trust in colleagues^*^	0.043 (−0.138, 0.223)	0.092	0.007	0.463	1.204	0.643
Burdened by increased workload^*^	−0.010 (−0.221, 0.200)	0.107	−0.002	−0.097	2.218	0.923
Burdened by change of duties^*^	0.331 (0.167, 0.496)	0.084	0.071	3.943	1.581	** <0.001**
Protective measures (national and local)^*^	0.196 (0.025, 0.367)	0.087	0.035	2.243	1.175	**0.025**

*IES-6, Impact of Event Scale; PHQ-2, Patient Health Questionnaire; GAD-2, Generalized Anxiety Module; ERI, Effort-Reward Imbalance Questionnaire; ESSI, ENRICHD Social Support Inventory*.

*Professional experience (1 ≤ 3 years, 2 = 3–6 years, 3 ≥ 6 years); degree of occupancy of the wards (1 = strongly below average, 2 = slightly below average, 3 = average, 4 = slightly above average, 5 = strongly above average), burden during COVID-19 (0 = not at all, 1 = little, 2 = middle, 3 = strong, 4 = very strong)*,

* *psychological characteristics and COVID-19 related work conditions (1 = strongly disagree, 2 = rather disagree, 3 = neither agree nor disagree, 4 = rather agree, 5 = strongly agree). significant predictors are marked in bold*.

## Discussion

The aim of the current cross-sectional web-based study was to examine the association between sociodemographic/ occupational, COVID-19-related, psychological variables, as well as work-related variables and PTSD symptomatology in a large sample of German HCW. Our findings revealed that an increased self-reported burden during the pandemic, increased fear of becoming infected or infecting relatives with the virus, sleep problems, feeling physically or mentally exhausted, as well as increased levels of depressiveness and generalized anxiety were associated with higher levels of PTSD symptomatology, with medium effects. Significant group differences also emerged for a range of other sociodemographic, occupational, and psychological variables, however only in a range of small or negligible effect sizes. According to regression analysis, the most relevant predictors for increased levels of PTSD symptoms included a higher level of depressiveness and generalized anxiety, increased fear of infecting relatives, as well as belonging to medical profession of MTA (compared to physicians).

Concerning sociodemographic variables, we confirmed that higher levels of PTSD were detected in female HCW, which is in line with the majority of previous studies in this context [e.g., (15, 18, 21)]. However, in the current sample, only a small effect size for gender was detected. A lack of a substantial group difference between men and women was also shown by our previous study with regard to depression and anxiety symptoms (10). Reasons for this may include advantageous working conditions for women in our sample, e.g., less frequent direct contact with infected patients (women: 50.3% vs. men: 54.3%, χ^2^ = 5.66, *p* = 0.017) or material (women: 49.2% vs. men: 57.6%, χ^2^ = 24.88, *p* < 0.001) as well as less displacement of the department (women: 12.9% vs. men: 15.6%, χ^2^ = 5.35, *p* = 0.021). In contrast to our hypothesis, our data provided evidence for higher PTSD scores in older age groups, albeit the difference was of a small effect size. While being in line with some studies [e.g., (20)], the majority of previous literature points toward higher PTSD symptoms in younger age groups [reviews see (15, 18)]. Importantly, the higher PTSD burden in older age groups did not emerge due to a higher frequency of MTA or nurses in these groups (≤40 years: 32.3% nurses, 35.1% MTA vs. >40 years: 22.7% nurses, 35.3% MTA). However, it is conceivable that this might be related to the fact that older HCW were more likely to belong to a risk group (≤40 years: 12.9% vs. >40 years: 41.4%, χ^2^ = 458.954, *p* < 0.001), which, in turn, was found to be related to higher PTSD symptoms.

When investigating group differences in PTSD severity with regards to work profession, the highest PTSD scores emerged in MTA as compared to nurses, physicians, and psychologists. Importantly, belonging to medical profession of MTA (compared to physicians and psychologists) turned out to be among the most relevant predictors for higher PTSD symptoms in the multivariate regression analysis. At first glance, this result seems to be at variance with previous findings observing elevated PTSD levels in nurses [e.g., (15, 18, 19, 24, 25)]. However, here, it is important to note that MTA are a largely neglected subgroup of HCW in previous literature. Importantly, the above result parallels findings of our previous study revealing the highest mental burden among MTA with regard to depressiveness, generalized anxiety, and stress reactions (e.g., insomnia and exhaustion) as compared to nurses and physicians (10). This pattern of findings also concurs with evidence of a study from Singapore revealing a three-fold increased general stress burden in non-medical HCW (e.g., allied health professionals, pharmacists, and technicians) as compared to physicians and nurses (38). Importantly, in Germany, MTA mainly work in the laboratory, but they have also responsibilities with direct patient contact within an inpatient and outpatient setting. Hence, it is conceivable that at the beginning of the pandemic, MTA were under high pressure to establish innovative tests and to increase testing capacity. This situation, in turn, may have contributed to increased uncertainty about possible infection with the sample material. Indeed, HCW who reported to have contact with contaminated material also reported higher PTSD levels and further analyses revealed that especially MTA reported to have significant more contact to contaminated material as compared to the other profession groups (MTA: 61.3%, physicians: 45.4%, nurses: 54.0%, psychologists: 2.4%, χ^2^ = 292.531, *p* < 0.001). Together, our data highlight that future studies as well as preventive programs should also target MTA. Likewise, this also relates to HCW working in an outpatient setting, which, in contrast to our hypothesis, also revealed significant higher PTSD scores than HCW working in a hospital, although the difference was of a negligible effect size.

In relation to COVID-19-related characteristics, higher PTSD levels were observed for HCW belonging to a risk group (because of age or previous illness) with a small effect size. This is in line with research showing higher prevalence rates of symptoms of stress, anxiety, and depression (39) as well as in our previous study also with a small effect (12). For those belonging to a risk group, the general fear of having a more severe course of the disease when getting infected might contribute to this association. In line with this assumption, HCW who belonged to a risk group stated a higher fear of becoming infected (rather or strongly agree: 37.7% of belonging to a risk group vs. 23.6% of not belonging to a risk group, χ^2^ = 142.127, *p* < 0.001), which, in turn, was found to be related to higher PTSD symptoms. Moreover, we have revealed in a previous study that these individuals showed fewer protective factors [e.g., social support and optimism; (12)]. Furthermore, negligible effects for higher PTSD symptoms were revealed for HCW who had contact with infected patients or with contaminated material and who were displaced of the department due to the pandemic. Especially, a lacking substantial group difference with regard to contact with infected patients contradicts previous evidence highlighting closeness to COVID-19 infected patients or working at the frontline as important risk factors for PTSD (15, 18, 19, 40). However, it is important to note that direct contact to Covid-19-infected patients also did not significantly contribute to higher depression or anxiety symptoms in our previous study (11). Remarkably, our data even revealed that HCW not working in patient care (irrespective of infection) even showed higher PTSD scores as compared to HCW being involved in patient care, albeit this effect was also negligible.

Concerning the effect of psychological variables on PTSD symptom severity, we revealed that a higher level of depressiveness and generalized anxiety were among the most relevant predictors for higher PTSD levels. This matches well with previous evidence [e.g., (18, 20)] and supports the notion that detrimental mental health status contributes to PTSD. However, due to the cross-sectional design of the current study, no conclusions can be drawn on whether PTSD symptoms contributed to increased comorbid depression and/or anxiety scores or whether depressive or anxious symptomatology were precursors of PTSD symptoms. Since the current study is part of a prospective web-based study, we have the great opportunity to investigate the sequelae of PTSD, depressive, and unspecific anxiety symptoms from the first to the second wave of the pandemic. Our study data further suggest higher PTSD levels to be associated with an increased burden during COVID-19, an increased fear of being infected or infecting relatives with the virus, sleep problems and a feeling of physical or mental exhaustion (medium effect size), as well as drinking more alcohol (small effect size). With regard to exhaustion, this finding seems to mesh well with data from a recent study among HCW, revealing a positive relationship between PTSD and burnout symptoms during COVID-19 (22). However, when interpreting the results, it should be considered that mental/physical exhaustion is highly associated with symptoms of depressiveness. Since our regression analyses showed depressiveness as a more relevant predictor, it is conceivable that the above effect is mainly attributable to higher levels of depressive symptoms in these individuals. Further prospective research is needed in this context in order to disentangle the effects of these variables on PTSD symptomatology.

With regard to fear of infection, our results are indirectly in line with data by Wang et al. (19) identifying not worrying about infection as a protective factor. An increased fear of becoming infected with the virus was also found as an important risk factor for generalized anxiety in our previous study (10). Importantly, according to regression analysis, an increased fear of infecting relatives was among the most relevant predictors for higher PTSD levels. It is conceivable that at the beginning of the COVID-19 pandemic fear of becoming infected or infecting relatives was a crucial influencing factor on PTSD symptoms since the exact pathways of infection were not clear and hygiene measures (e.g., regularly wearing of mouth-nose masks) or regularly testing procedures were not established yet. The current finding of sleeping difficulties to contribute to higher PTSD symptoms is in line with previous studies [e.g., (18, 21)]. Hence, this aspect may also be relevant to consider in future preventive approaches. In line with previous evidence [e.g., (15, 18, 22)], an increased level of social support contributed to lower PTSD levels in our sample, albeit this effect was only small. Study data by our group indicate that social support seems to be more relevant to predict lower depression and generalized anxiety scores (11).

Our data revealed only small positive relationships between PTSD symptoms and work-related characteristics including increased chronic work-related stress (i.e., imbalance of high efforts spent and low rewards received), recovery during leisure time, trust in colleagues, and burden by the increase of work load as well as by the change in duties. While these findings commensurate well with a study by Wang et al. (41) showing that low job satisfaction was a risk factor for PTSD among nurses, our results do not support a relevant impact of these variables as compared to the above psychological variables.

### Limitations

Limitations of the current study include the cross-sectional design of the study precluding the examination of causal relationships. However, as mentioned above, future studies will be published examining prospective relationships between the above variables and developing PTSD symptoms. Furthermore, it is important to mention that the survey was conducted at the beginning of the pandemic when the impact on healthcare systems was relatively low in Germany, which, in turn, may have resulted in reduced variance in PTSD symptomatology. Again, analyses of the data obtained during the second wave of the pandemic, which co-occurred with higher occupancy rates and intensive care supplies, might be instructive. Further, since the survey was primarily conducted in selected university hospitals, it is conceivable that these were not representative of the COVID-19 situation at that time throughout Germany. Another limitation concerns the voluntary nature of our study, which may be related to a response bias. Specifically, it is conceivable that non-respondents were either too symptomatic or were too relaxed and thus did not participate in the current survey. Our HCW sample consisted of a larger proportion of women. However, here, it is important to note that regarding university hospitals where the largest proportion of respondents were achieved, we were able to show that the study sample (i.e., physicians, nurses, and MTA) showed a representative gender proportion (10). Given that a brief measure for the assessment of PTSD symptomatology was used (i.e., IES-6), no information on cut-off scores were available, precluding the possibility to calculate the exact prevalence rate of PTSD in the current sample. Furthermore, it is important to note that for the IES-6, we revealed a Cronbach's alpha value bordering on a level defined as acceptable. A further limitation is related to the fact that no detailed information on trauma exposure during and before the COVID-19 pandemic were obtained. This is of relevance considering previous evidence supporting the notion that trauma history has an impact on the severity of PTSD symptoms [e.g., (42)]. Finally, while the use of self-report questionnaires provides a range of advantages (e.g., financial costs and large sample), limitations of these measures should be considered [e.g., response bias; (43)].

### Integration of Findings and Implications

Given the large sample size of the current study, we observed significant associations for a range of sociodemographic/occupational, COVID-19-related, psychological, and work-related variables. However, most of the associations were in a range of small or even negligible effect sizes, except for various psychological variables reflecting poorer mental health with moderate effect sizes. Based on multivariate regression analysis including all variables of interest, higher levels of depressiveness and generalized anxiety as well as increased fear of infecting relatives in addition to a working role as MTA (compared to physicians) were revealed as most relevant predictors of PTSD symptomatology. Based on the correlates and risk factors for PTSD symptomatology identified by the current study, the following preventive and interventive possibilities might be of relevance: Targeted training to perceive and regulate distress levels including symptoms of depression and generalized anxiety and particularly sleep difficulties as well as physical and mental exhaustion might be helpful to ameliorate PTSD symptomatology among HCW. The fear of infecting relatives with the virus might be reduced by psychoeducative information on infection, transparent hygiene measures, as well as regular testing. Importantly, our data suggest that MTA exhibited increased levels of PTSD symptoms, especially as compared to physicians and psychologists. This finding highlights the need to specifically also include indispensable professionals working more in the background besides HCW in the frontline in the context of research and the development of preventive or interventive programs.

## Data Availability Statement

The raw data supporting the conclusions of this article will be made available by the authors, without undue reservation.

## Ethics Statement

The studies involving human participants were reviewed and approved by Ethics Committee of the Medical Faculty of the Friedrich-Alexander University Erlangen-Nürnberg (FAU) and registered on ClinicalTrials (DRKS-ID: DRKS00021268). The patients/participants provided their written informed consent to participate in this study.

## Author Contributions

YE, EM, FG, PB, and LJ-B designed the study and wrote the protocol. YE, EM, FG, PB, LJ-B, CA, NH, SS-S, and KW conducted the study. SS-S and LS analyzed the data and wrote the manuscript. KW, YE, EM, FG, LJ-B, PB, CA, and NH provided critical revision of the manuscript. All authors contributed to and have approved the final manuscript.

## Conflict of Interest

The authors declare that the research was conducted in the absence of any commercial or financial relationships that could be construed as a potential conflict of interest.

## Publisher's Note

All claims expressed in this article are solely those of the authors and do not necessarily represent those of their affiliated organizations, or those of the publisher, the editors and the reviewers. Any product that may be evaluated in this article, or claim that may be made by its manufacturer, is not guaranteed or endorsed by the publisher.

## References

[1] Arvidsdotter T Marklund B Kylen S Taft C Ekman I. Understanding persons with psychological distress in primary health care. Scand J Caring Sci. (2016) 30:687–94. 10.1111/scs.12289 26463897

[2] Krishnamoorthy Y Nagarajan R Saya GK Menon V. Prevalence of psychological morbidities among general population, healthcare workers and COVID-19 patients amidst the COVID-19 pandemic: a systematic review and meta-analysis. Psychiatry Res. (2020) 293:113382. 10.1016/j.psychres.2020.113382 32829073 PMC7417292

[3] Xiong J Lipsitz O Nasri F Lui LMW Gill H Phan L. Impact of COVID-19 pandemic on mental health in the general population: a systematic review. J Affect Disord. (2020) 277:55–64. 10.1016/j.jad.2020.08.001 32799105 PMC7413844

[4] Cenat JM Blais-Rochette C Kokou-Kpolou CK Noorishad PG Mukunzi JN McIntee SE. Prevalence of symptoms of depression, anxiety, insomnia, posttraumatic stress disorder, and psychological distress among populations affected by the COVID-19 pandemic: a systematic review and meta-analysis. Psychiatry Res. (2021) 295:113599. 10.1016/j.psychres.2020.113599 33285346 PMC7689353

[5] Bäuerle A Steinbach J Schweda A Beckord J Hetkamp M Weismuller B. Mental health burden of the COVID-19 outbreak in Germany: predictors of mental health impairment. J Prim Care Community Health. (2020) 11:2150132720953682. 10.1177/2150132720953682 32865107 PMC7457643

[6] Petzold MB Bendau A Plag J Pyrkosch L Mascarell Maricic L Betzler F. Risk, resilience, psychological distress, and anxiety at the beginning of the COVID-19 pandemic in Germany. Brain Behav. (2020) 10:e01745. 10.1002/brb3.1745 32633464 PMC7361063

[7] Pappa S Ntella V Giannakas T Giannakoulis VG Papoutsi E Katsaounou P. Prevalence of depression, anxiety, and insomnia among healthcare workers during the COVID-19 pandemic: a systematic review and meta-analysis. Brain Behav Immun. (2020) 88:901–7. 10.1016/j.bbi.2020.05.026 32437915 PMC7206431

[8] Luo M Guo L Yu M Jiang W Wang H. The psychological and mental impact of coronavirus disease 2019 (COVID-19) on medical staff and general public - a systematic review and meta-analysis. Psychiatry Res. (2020) 291:113190. 10.1016/j.psychres.2020.113190 32563745 PMC7276119

[9] Salazar de Pablo G Vaquerizo-Serrano J Catalan A Arango C Moreno C Ferre F. Impact of coronavirus syndromes on physical and mental health of health care workers: systematic review and meta-analysis. J Affect Disord. (2020) 275:48–57. 10.1016/j.jad.2020.06.022 32658823 PMC7314697

[10] Morawa E Schug C Geiser F Beschoner P Jerg-Bretzke L Albus C. Psychosocial burden and working conditions during the COVID-19 pandemic in Germany: The VOICE survey among 3678 health care workers in hospitals. J Psychosom Res. (2021) 144. 10.1016/j.jpsychores.2021.110415 33743398 PMC7944879

[11] Beschoner P Jarczok M Kempf M Weimer K Geiser F Erim Y. EgePan-Studie zur psychosozialen Belastung durch die Covid-19-Pandemie - MTAs die vergessene Berufsgruppe. under review. 10.13109/zptm.2021.67.oa15 34889716

[12] Schug C Morawa E Geiser F Hiebel N Beschoner P Jerg-Bretzke L. Social support and optimism as protective factors for mental health among 7765 healthcare workers in Germany during the COVID-19 pandemic: results of the VOICE study. Int J Environ Res Public Health. (2021) 18:3827. 10.3390/ijerph18073827 33917493 PMC8038794

[13] Bridgland VME Moeck EK Green DM Swain TL Nayda DM Matson LA. Why the COVID-19 pandemic is a traumatic stressor. PLoS ONE. (2021) 16:e0240146. 10.1371/journal.pone.0240146 33428630 PMC7799777

[14] Association AP. Diagnostic and Statistical Manual of Mental Disorders (DSM-5^®^). Arlington, VA: American Psychiatric Pub (2013).

[15] Carmassi C Foghi C Dell'Oste V Cordone A Bertelloni CA Bui E. PTSD symptoms in healthcare workers facing the three coronavirus outbreaks: what can we expect after the COVID-19 pandemic. Psychiatry Res. (2020) 292:113312. 10.1016/j.psychres.2020.113312 32717711 PMC7370915

[16] Agberotimi SF Akinsola OS Oguntayo R Olaseni AO. Interactions between socioeconomic status and mental health outcomes in the Nigerian context amid COVID-19 pandemic: a comparative study. Front Psychol. (2020) 11:559819. 10.3389/fpsyg.2020.559819 33117227 PMC7573662

[17] Chen B Li QX Zhang H Zhu JY Yang X Wu YH. The psychological impact of COVID-19 outbreak on medical staff and the general public. Curr Psychol. (2020) 1–9. 10.21203/rs.3.rs-21213/v1. [Epub ahead of print]. 33046955 PMC7539749

[18] Yuan K Gong YM Liu L Sun YK Tian SS Wang YJ. Prevalence of posttraumatic stress disorder after infectious disease pandemics in the twenty-first century, including COVID-19: a meta-analysis and systematic review. Mol Psychiatry. (2021) 1–17. 10.1038/s41380-021-01036-x. [Epub ahead of print]. 33542468 PMC7861006

[19] Wang Y Ma S Yang C Cai Z Hu S Zhang B. Acute psychological effects of Coronavirus Disease 2019 outbreak among healthcare workers in China: a cross-sectional study. Transl Psychiatry. (2020) 10:348. 10.1038/s41398-020-01031-w 33051440 PMC7552583

[20] Li X Li S Xiang M Fang Y Qian K Xu J. The prevalence and risk factors of PTSD symptoms among medical assistance workers during the COVID-19 pandemic. J Psychosom Res. (2020) 139:110270. 10.1016/j.jpsychores.2020.110270 33070044 PMC7536549

[21] Blekas A Voitsidis P Athanasiadou M Parlapani E Chatzigeorgiou AF Skoupra M. COVID-19: PTSD symptoms in Greek health care professionals. Psychol Trauma. (2020) 12:812–9. 10.1037/tra0000914 32853011

[22] Johnson SU Ebrahimi OV Hoffart A. PTSD symptoms among health workers and public service providers during the COVID-19 outbreak. PLoS ONE. (2020) 15:e0241032. 10.1371/journal.pone.0241032 33085716 PMC7577493

[23] Benfante A Di Tella M Romeo A Castelli L. Traumatic stress in healthcare workers during COVID-19 pandemic: a review of the immediate impact. Front Psychol. (2020) 11:569935. 10.3389/fpsyg.2020.569935 33192854 PMC7645025

[24] Di Tella M Benfante A Castelli L Romeo A. Anxiety, depression, and posttraumatic stress in nurses during the COVID-19 outbreak. Intensive Crit Care Nurs. (2021) 64:103014. 10.1016/j.iccn.2021.103014 33589343 PMC7857052

[25] Jo SH Koo BH Seo WS Yun SH Kim HG. The psychological impact of the coronavirus disease pandemic on hospital workers in Daegu, South Korea. Compr Psychiatry. (2020) 103:152213. 10.1016/j.comppsych.2020.152213 33096399 PMC7553934

[26] Thoresen S Tambs K Hussain A Heir T Johansen VA Bisson JI. Brief measure of posttraumatic stress reactions: impact of Event Scale-6. Soc Psychiatry Psychiatr Epidemiol. (2010) 45:405–12. 10.1007/s00127-009-0073-x 19479171

[27] Maercker A Schützwohl M. Erfassung von psychischen Belastungsfolgen: Die Impact of Event Skala-revidierte Version (IES-R). Diagnostica. (1998) 44:130–41. 10.1037/t55092-000

[28] Löwe B Wahl I Rose M Spitzer C Glaesmer H Wingenfeld K. A 4-item measure of depression and anxiety: validation and standardization of the Patient Health Questionnaire-4 (PHQ-4) in the general population. J Affect Disord. (2010) 122:86–95. 10.1016/j.jad.2009.06.019 19616305

[29] Kendel F Spaderna H Sieverding M Dunkel A Lehmkuhl E Hetzer R. Eine deutsche Adaptation des ENRICHD Social Support Inventory (ESSI). Diagnostica. (2011) 57:99–106. 10.1026/0012-1924/a000030

[30] The, ENRICHD, Investigators. Enhancing recovery in coronary heart disease patients (ENRICHD): study design and methods. The ENRICHD investigators. Am Heart J. (2000) 139:1–9. 10.1016/S0002-8703(00)90301-6 10618555

[31] Matsuishi K Kawazoe A Imai H Ito A Mouri K Kitamura N. Psychological impact of the pandemic (H1N1) 2009 on general hospital workers in Kobe. Psychiatry Clin Neurosci. (2012) 66:353–60. 10.1111/j.1440-1819.2012.02336.x 22624741

[32] Siegrist J Starke D Chandola T Godin I Marmot M Niedhammer I. The measurement of effort-reward imbalance at work: European comparisons. Soc Sci Med. (2004) 58:1483–99. 10.1016/S0277-9536(03)00351-4 14759692

[33] Rugulies R Aust B Madsen IE. Effort-reward imbalance at work and risk of depressive disorders. A systematic review and meta-analysis of prospective cohort studies. Scand J Work Environ Health. (2017) 43:294–306. 10.5271/sjweh.3632 28306759

[34] Eddy P Wertheim EH Kingsley M Wright BJ. Associations between the effort-reward imbalance model of workplace stress and indices of cardiovascular health: a systematic review and meta-analysis. Neurosci Biobehav Rev. (2017) 83:252–66. 10.1016/j.neubiorev.2017.10.025 29111334

[35] Weigl M Schneider A Hoffmann F Angerer P. Work stress, burnout, and perceived quality of care: a cross-sectional study among hospital pediatricians. Eur J Pediatr. (2015) 174:1237–46. 10.1007/s00431-015-2529-1 25846697

[36] Siegrist J Li J Montano D. Psychometric Properties of the Effort-Reward Imbalance Questionnaire. Department of Medical Sociology, Faculty of Medicine, Duesseldorf University, Germany (2014).31572259

[37] Cohen J. Statistical Power Analysis for the Behavioral Sciences. Cambridge, MA: Academic Press (2013).

[38] Tan BYQ Chew NWS Lee GKH Jing M Goh Y Yeo LLL. Psychological impact of the COVID-19 pandemic on health care workers in Singapore. Ann Intern Med. (2020) 173:317–20. 10.7326/M20-1083 32251513 PMC7143149

[39] Sayeed A Kundu S Al Banna MH Christopher E Hasan MT Begum MR. Mental health outcomes of adults with comorbidity and chronic diseases during the COVID-19 pandemic: a matched case-control study. Psychiatr Danub. (2020) 32:491–8. 10.31234/osf.io/qh6b5 33370758

[40] Bohlken J Schomig F Lemke MR Pumberger M Riedel-Heller SG. COVID-19 pandemic: stress experience of healthcare workers - a short current review. Psychiatr Prax. (2020) 47:190–7. 10.1055/a-1159-5551 32340048 PMC7295275

[41] Wang YX Guo HT Du XW Song W Lu C Hao WN. Factors associated with post-traumatic stress disorder of nurses exposed to corona virus disease 2019 in China. Medicine (Baltimore). (2020) 99:e20965. 10.1097/MD.0000000000020965 32590808 PMC7328992

[42] Kessler RC Aguilar-Gaxiola S Alonso J Bromet EJ Gureje O Karam EG. The associations of earlier trauma exposures and history of mental disorders with PTSD after subsequent traumas. Mol Psychiatry. (2018) 23:1892–9. 10.1038/mp.2017.194 28924183 PMC5858954

[43] Demetriou C Ozer BU Essau CA. Self-report questionnaires. Encyclopedia Clin Psychol. (2014) 1–6. 10.1002/9781118625392.wbecp507. [Epub ahead of print].

